# 3D CRISPR screen in prostate cancer cells reveals PARP inhibitor sensitization through TBL1XR1-SMC3 interaction

**DOI:** 10.3389/fonc.2022.999302

**Published:** 2022-11-29

**Authors:** Huan Zhang, Huanyao Gao, Yayun Gu, August John, Lixuan Wei, Minhong Huang, Jia Yu, Adeyemi A. Adeosun, Richard M. Weinshilboum, Liewei Wang

**Affiliations:** ^1^ School of Medicine, Nantong University, Nantong, China; ^2^ Division of Clinical Pharmacology, Department of Molecular Pharmacology and Experimental Therapeutics, Mayo Clinic, Rochester, MN, United States

**Keywords:** PARP inhibitor, prostate cancer, CRISPR screen, TBL1XR1, SMC3, γH2AX

## Abstract

Poly(ADP-ribose) (PAR) polymerase inhibitors (PARPi) either have been approved or being tested in the clinic for the treatment of a variety of cancers with homologous recombination deficiency (HRD). However, cancer cells can develop resistance to PARPi drugs through various mechanisms, and new biomarkers and combination therapeutic strategies need to be developed to support personalized treatment. In this study, a genome-wide CRISPR screen was performed in a prostate cancer cell line with 3D culture condition which identified novel signals involved in DNA repair pathways. One of these genes, TBL1XR1, regulates sensitivity to PARPi in prostate cancer cells. Mechanistically, we show that TBL1XR1 interacts with and stabilizes SMC3 on chromatin and promotes γH2AX spreading along the chromatin of the cells under DNA replication stress. TBL1XR1-SMC3 double knockdown (knockout) cells have comparable sensitivity to PARPi compared to SMC3 knockdown or TBL1XR1 knockout cells, and more sensitivity than WT cells. Our findings provide new insights into mechanisms underlying response to PARPi or platin compounds in the treatment of malignancies.

## Introduction

Poly(ADP-ribose) polymerase (PARP) inhibitors (PARPi) are primarily used in cancer patients with loss-of-function BRCA1- or BRCA2 since these tumors are deficient in the homologous recombination (HR) DNA repair pathway, which carry out error-free repair of DNA double-strand breaks (DSBs) during the S and G2 phases of the cell cycle (1, 2). PARPi have been approved for the treatment of HR-defective breast, ovarian, pancreatic and prostate cancers and is undergoing clinical trials for other cancer types (3). PARP1 is the most abundant protein in PARP family and is involved in various cellular processes, including multiple DNA damage repair pathways, stabilization of DNA replication forks, and chromatin remodeling by post-translationally attaching poly(ADP-ribose) (PAR) to multiple target proteins and to itself (4). During the process of DNA base excision repair (BER), PARP1 is rapidly recruited to damaged sites to promote recruitment and PARylation of XRCC1, which acts as a scaffold for the recruitment of PNKP, APTX and LIG3 to process single-strand break (SSB) repair (5, 6). Once DNA repair is initiated, AutoPARylation of PARP1 enhances its release from DNA, an essential step for various DNA repair processes (7).

PARPi not only impairs DNA repair by inhibiting the catalytic activity of PARP to cause synthetic lethality in HR-deficient cells but also enhances cytotoxicity effect in cells through PARP trapping (8, 9). PARPi inhibit the PARylation of PARP itself and prevent its release from the SSB sites of DNA, thereby forming stable PARP-DNA complexes (8, 10, 11). The PARP-DNA complexes block DNA replication during the S phase of the cell cycle, causing DNA double-strand breaks and replication fork collapse, leading to cell death.

Tumor cells generate resistance to PARPi through several mechanisms. As a key protein of the NHEJ pathway, 53BP1 inhibits the HR pathway by competing with BRCA1-CtIP for binding to DSBs and preventing DNA ends from being digested into single strands (12). Defectiveness of the 53BP1-RIF1-REV7 complex restores DNA end resection in BRCA1-deficient cells, thereby restoring HR repair function leading to PARPi resistance (13, 14). The accumulation of RAD51-ssDNA filament is also an important reason for restoring HR repair to produce PARPi resistance (15, 16). PARPi enhances cytotoxicity through PARP trapping and therefore, downregulation or inactivation of PARP protein caused by mutations can lead to PARPi resistance (17). In various cancers, a secondary mutation in the BRCA protein re-establishes the original reading frame of the BRCA protein to restore its function and confers resistance to PARPi-based therapy (18–20). During DNA replication, PARP1 and BRCA1/2 protect stalled replication forks from nuclease (i.e., MRE11, DNA2, MUS81) digestion, which will induce genome instability and cell death (21). Defection in PTIP, MELL3/4 and CHD4 prevent the recruitment of MRE11 nucleases to the stalled replication forks resulting in PARPi resistance in BRCA-deficient cells (22). Recently, LIG3 had been reported to confer PARPi resistance in BRCA1/53BP1 double-deficient cells by preventing the formation of MRE11-mediated post-replicative ssDNA gaps (23).

To further understand better of PARPi response in prostate cancer, in this study, we performed a genome-wide CRISPR screen in prostate cancer cells with 3D culture condition and identified TBL1XR1 as a factor regulating sensitivity to PARPi in prostate cancer cells. TBL1XR1 is a subunit of NCOR1/SMAT complex (24), which are among the first identified nuclear receptor corepressors (25, 26). TBL1XR1 has been reported to be associated with tumorigenesis, metastases, chemoresistance and poor overall survival in several cancers (27–32). Here, we found that TBL1XR1 deficiency sensitizes prostate cancer cells to PARPi through decreasing γH2AX foci formation near the DNA damage sites during S phase of the cell cycle. Immunoprecipitation-mass spectrometry (IP-MS) and co-immunoprecipitation (co-IP) assay showed that TBL1XR1 directly interacted with cohesin subunit SMC3, and nucleus fractionation assay demonstrated its critical role in the stabilization of SMC3 chromatin-bound status, a process promoting the spreading of γH2AX along the chromosome near the DNA damage sites (33).

## Materials and methods

### Cell culture

22RV1 cells purchased from ATCC were routinely cultured in RPMI1640 medium (Gibco, Grand Island, NY) supplemented with 10% FBS (Atlanta Biologicals, Flowery Branch, GA). To develop olaparib resistant cell line, cells were maintained in the medium supplemented with 10μM of olaparib (Selleck Chemicals, Houston, TX) for 3 months until the viability reached over 95%.

293T cell line (Lenti-X 293T Cell Line) (34) was ordered from TaKaRa (Cat. No. 632180) cultured by DMEM medium supplemented with 10% Fetal Bovine Serum (FBS). All the cells were cultured at 37°C in 5% CO_2_ incubator.

For 3D culture, we used Nunclon™ Sphera™ Dishes (Thermo Scientific, Cat. No. 174945) and added methylcellulose (0.5%; Fisher, Cat No. M-352) in RPMI 1640 growth medium supplemented 10% FBS to prevent excessive aggregation of cells in spheroid culture and to maintain even spheroid size.

### Lentiviral packaging

CRISPR Cas9 Expression Construct (pRCCBla-CMV-Cas9-2A-Blast) was ordered from Cellecta (Cat. No. SVC9B-PS). CRISPR Human Genome 80K Knockout Library (total 77,736 sgRNAs and 4 sgRNA to each of the ~19,000 protein-coding genes) was ordered from Cellecta (Cat. No. KOHGW-80K-P). The LentiPrep™ Lentiviral Reagent (Cellecta, Cat. No. LTSET-G) was used for packaging and transduction. Lentiviral packaging was performed according to “CRISPR Pooled Lentiviral sgRNA Libraries Manual” from Cellecta. The filtered virus-containing medium was aliquoted and stored in -80°C freezer for future transduction.

### Lentiviral transduction

For making Cas9-expressing cells, 22RV1 cells were transduced by Cas9 Expression lentivirus with 1 μL/mL LentiTran Transduction Reagent (Cellecta, Cat. No. LTDR1). 10μg/mL Blasticidin was supplemented to medium for antibiotic selection and cells were grown under selection for 2 weeks to generate stable Cas9 expressing 22RV1 cells (22RV1_Cas9). For making library knockout (KO) cells, 22RV1_Cas9 cells were transduced with library virus using 1 μL/mL LentiTran Transduction Reagent, and the MOI is about 0.3. 0.8μg/mL puromycin was used for antibiotic selection and cells were grown under the selection for 5 days to generate 22RV1 KO library cells (22RV1-KOlib). Two replicates of 4×10^7^ library cells were used for DNA extraction and setup as base-line (input).

### CRISPR screen

For genome-wide CRISPR screen in 22RV1 cells, 4×10^7^ library cells were seeded in 10 Nunclon™ Sphera™ Dishes (4×10^6^ cells per dish) for each treatment in duplicates. The next day, the medium was replaced with fresh medium either with 0.1% DMSO (Vehicle Control), or 3μM olaparib. The medium with drug or vehicle was refreshed every other day. On the 8th day of treatment, all cells were harvested and stored in -80°C freezer.

The DNA of cells was extracted using the phenol/chloroform method. The fragments that inserted into genome containing sgRNAs were amplified by KAPA HiFi HotStart Readyix (Roche, Cat. No. KK2602). Primers are listed as following: SeqF- TGAAAGTATTTCGATTTCTTGGC, SeqR- TCAThe raw sequencing data in fastq was quality checked by FastQC. The vector backbone and sequencing adaptor was trimmed by BBduk, and the resulted 20mers were mapped to the sgRNA sequence library file provided by the vendor (Cellecta) by bowtie2 (35). After removal of low-map quality reads (MAPQ<30), raw counts of sgRNAs were called by Mageck count function in Mageck package (36). sgRNAs with less than 200 counts were excluded from further analysis. Knockout efficiency was estimated using the Spacer Scoring for CRISPR (SSC) (37). sgRNA enrichment after olaparib treatment was then calculated using Mageck mle function in the Mageck package (36). We defined significance as abs(beta) ≥ 0.2 and false discovery rate (FDR) ≤ 0.05 comparing olaparib vs vehicle treated samples, and excluded genes if it met any of the following criteria: a) has only 1 or 2 sgRNA remained after previous step; b) mean counts of all sgRNA in vehicle-treated samples are less than 512; or c) significantly viability essential, as defined by beta ≤ -0.2 and FDR ≤ 0.05 comparing vehicle treatment and input (**Supplementary Data 3**). The Volcano plot was drawn with the beta score (indicates the degree of selection, similar to the term log-fold change) and z score (a numerical measurement that describes a value’s relationship to the mean of a group of values).

### CRISPR knock out

For knocking out candidate genes, several double-sgRNA vectors were constructed based on pRSG16-U6-sg-UbiC-TagRFP-2A-Puro. The primers are listed in **Supplementary Table 1**. The vectors were packaged and the lentivirus was transduced into 22RV1_CAS9 cells as described above. The cells were treated by 1μg/mL puromycin for 7 days to select knock-out pool cells.

To generate monoclonal knockout cell lines of TBL1XR1 in parental and olaparib-resistant 22RV1 cells (OalR), SpCas9 Nuclease (Cat. No. 1081058), tracrRNA (Cat. No. 1075927) and two crRNAs for TBL1XR1 (**Supplementary Table 1**) were purchased from IDT (Coralville, Iowa) and the genome editing was performed according to the “Alt-R CRISPR-Cas9 system—RNP transfections” protocol from IDT using the Lipofectamine™ CRISPRMAX™ Cas9 Transfection Reagent (Invitrogen, Cat. No. CMAX00001). The TBL1XR1 protein level in the knockout cell lines derived from monoclonal was validated by western blot analysis.

### Cytotoxicity assay

24-h before drug treatment, cells were seeded in 96-well plates at 5000 cells per well density and treated by various doses of drug after overnight culturing. The cells were treated for 4 days before viability being examined by CyQUANT™ Direct assay (Invitrogen, Cat. No. C35011). For 3D culture cytotoxicity assay, cells were seeded in Sphera Low-Attachment 96-well plates (Thermo Scientific, Cat. No. 174927) at 5000 cells per well density with 3D culture medium and treated by various doses of drug 24h later. The cells were treated for 6 days before viability being examined by CellTiter-Glo^®^ 3D Cell Viability Assay (Promega, Cat. No. G9683).

### Colony formation assay

500 cells per well were seeded in 6-well plates and cultured overnight. Subsequently, the cells were treated with different concentrations of olaparib for two weeks, and the medium and drug were refreshed on day 7. The colonies were fixed by methanol for 30 mins and stained by Crystal Violet (MilliporeSigma, Cat. No. 65092A-95) overnight, followed by washing with ddH_2_O several times until the background was clear.

### Plasmids and RNA interference

cDNA for human TBL1XR1 was ordered from Sino Biological (Cat. No. HG20220-UT). The cDNA fragment was amplified by primers (**Supplementary Table 1**) and inserted into linearized pWPXL plasmid (Addgene, Plasmid #12257) by In-fusion Kit (TaKaRa, Cat. No. 638945). Plasmid transfections were performed with Lipofectamine™ 2000 Transfection Reagent (Invitrogen, Cat. No. 11668030). siRNAs of PARP1 (Cat. No. M-006656-01) and SMC3 (Cat. No. M-006834-01) were purchased from Horizon Discovery. siRNA transfections were performed with Lipofectamine™ RNAiMAX Transfection Reagent (Invitrogen, Cat. No. 13778075).

### Protein extraction and western blot

Western blot was performed using cells collected for the following experiments: For time course of drug treatment, the cells were treated with 10μM laparib and harvested with RIPA buffer at 0h (without treatment), 1h, 2h and 4h after being washed by ice-cold PBS. For detecting the soluble nuclear and chromatin-bound proteins in cells, the cells were treated by laparib for 4h and performed protein extraction by Subcellular Protein Fractionation Kit (Thermo Scientific, Cat. No. 78840).

The protein was extracted by RIPA buffer (Thermo Scientific, Cat. No. 89901) with protease inhibitor (Roche, Cat. No. 11873580001) and phosphatase inhibitor (Roche, Cat. No. 4906845001). Protein concentrations were assessed using the BCA assay (Thermo Scientific, Cat. No. 23227).

Proteins generated from both procedures were detected by Western blot according to standard protocols using ChemiDoc™ Touch Imaging System (Bio-Rad). A list of all primary antibodies used in the western blot experiments is provided in **Supplementary Table 2**.

### DNA fiber assay

This assay was performed as previously described (38). Cells were seeded in 6-well plates treated by vehicle or 10μM laparib for 200min and then added 10μM IdU incubated for 20min. Following washing, 250μM CldU was added and incubated for additional 20min. The cells were released from the plate by trypsin and washed by ice-cold PBS twice. Subsequently, cells were resuspended in PBS and diluted to 5×10^5^ cells/mL. The cell suspension was plated on microscope slide and lysed with spreading buffer. Individual DNA fibers were released and spread by tilting the slides at 25-40 degrees. After air-drying, fibers were fixed by 3:1 methanol/acetic acid at room temperature for 10 min. After air-drying again, fibers were washed twice in ddH_2_O, denatured with 2.5M HCl for 1h, washed twice with PBS, and blocked with blocking buffer (PBS + 3%BSA) for 1 hr. Next, slides were incubated overnight at 4°C with primary antibodies (**Supplementary Table 2**) diluted in blocking buffer. The next day, the slides were washed twice with PBS and incubated with secondary antibodies for 1h. After washing and air-drying, the slides were mounted with mounting medium (Abcam, Cat. No. ab104139). Finally, visualization of green and/or red signals (measure at least 200 fibers for each experiment) was captured by confocal microscopy (Zeiss LSM 780).

### Micronucleus assay

Cells were treated with vehicle, 3μM or 6μM laparib for 48h, then fixed by 4% PFA and washed twice by ddH_2_O. After air-drying, the cells were mounted by mounting medium with DAPI staining. Imagines (5 fields for each experiment and at least 200 cells in each field) were generated by confocal microscopy (Zeiss LSM 780).

### Comet assay

Comet assay was performed based on the manufacturer’s instruction (R&D systems 4250-050-K). Briefly, 3×10^5^ 22RV1 or TBL1XR1-knockout cells were seeded in 6 wells. The following day, cells were treated with 5μM laparib. After 48h, cells were harvested by trypsinization and diluted to 2×10^5^ cells/mL in cold PBS. Cells were mixed with melted LMAgarose at 1:10 ratio, and 50μL were immediately transferred to CometSlide, and cool at 4°C for 30min, and submerged in lysis solution for 2 hours in dark. For neutral condition, slides were immersed in neutral buffer (100mM Tris, 300mM NaAc, pH9.0) for 30min before electrophoresis at 21V for 45min. DNA was then precipitated in precipitation buffer (1M NH_4_Ac in 85% Ethanol) for 30min, followed by 70% Ethanol for 30min, before drying at 37°C and stained by SYBR Gold (Invitrogen). For alkaline condition, after lysis, slides were immersed in alkaline buffer (200mM NaOH, 1mM EDTA, pH>13) for 30min at RT, before electrophoresis at 21V for 30min. Slides were washed twice with H_2_O, followed by 70% Ethanol for 5min, before drying at 37°C and stained by SYBR Gold. Images were captured by Nikon Ti80 fluorescent microscope and comet tail moments were quantified using CaspLab. At least 50 cells were counted, and the comparison was performed using student’s t-test.

### EdU staining and immunofluorescence

Cells were treated for 3h 40min with vehicle or 10μM olaparib and then incubated with 10μM EdU for 20min. EdU staining was performed with the Click-iT™ EdU Cell Proliferation Kit (Invitrogen, Cat. No. C10339). After staining with the Alexa Fluor™ 594 dye, the cells were blocked with 3% BSA in PBS for 1h and then incubated with γH2AX AF488 Conjugate Antibody (MilliporeSigma, Cat. No. 05-636-AF488) for 1h. Next, the cells were washed twice by PBS and mounted by mounting medium with DAPI. The visualization of cells with EdU (Red), γH2AX foci (Green) and Nucleus (Blue) was captured by confocal microscopy (Zeiss LSM 780). The foci number of γH2AX foci per cell was counted by ImageJ software.

### Immunoprecipitation-mass spectrometry

For IP-MS experiments, 1×10^7^ cells were seeded in a 15-cm dish for each replicate. There were duplicate samples for both 22RV1 and TBL1XR1 KO cells. After culturing overnight, the cells were scraped and washed twice with ice-cold PBS. Then the cell pellets were resuspended and lysed with the Pierce™ IP Lysis Buffer (Thermo Scientific, Cat. No. 87788) containing protease inhibitor and phosphatase inhibitor. After protein concentrations were assessed, 7500μg total lysate of each sample was incubated with 100μL TBL1XR1 antibody at 4°C overnight. The next day, 300μL Pierce™ Protein A/G Magnetic Beads (Thermo Scientific, Cat. No. 88803) were added into each sample and incubated for 1h at room temperature. The magnetic beads were washed four times with the lysis buffer and eluted with the SDS buffer. The eluted proteins of samples were then loaded on SDS-PAGE and separated. The gel was stained by Coomassie Blue and cut into several pieces then sent to Harvard for Mass Spectrometry.

### Co-immunoprecipitation

For co-IP experiments, procedures were similar to those described in IP-MS but with decreased cells, antibodies and magnetic beads. For 22RV1 and TBL1XR1 KO cells, 3×10^6^ cells were cultured for 24h before being treated by vehicle or 10μM olaparib. After 4h treatment, the cells were harvested, lysed and incubated with 30μL magnetic beads and10μL TBL1XR1 antibody overnight. After washing, the eluted proteins were separated using SDS-PAGE and electro-transferred to PVDF membranes for western blot. For transient transfection of TBL1XR1-GFP and empty GFP, 48h after transfection, the 293T cells were treated with vehicle or 100μM olaparib for 4h. The lysate was incubated with GFP antibody and GFP magnetic beads (MBL, Cat. NO. D153-11). A list of all primary antibodies used in the experiments is provided in **Supplementary Table 2**.

### Pathway analysis

Pathway analysis of CRISPR screen and IP-MS data was performed by Erichr portal(https://maayanlab.cloud/Enrichr/) and download the results from BioPlanet 2019 database set (39).

### Survival analysis

The survival analysis was performed on KM-plotter (http://kmplot.com/analysis/) ovarian cancer cohorts using progression-free survival as primary outcome (40). Only samples within the treatment group containing platin were included in the analysis. Samples were grouped by expression of TBL1XR1 (probes 221428_s_at and 222633_at) or SMC3 (probes 209258_s_at and 209259_s_at) at optimal cutoff automatically determined by kmplotter, and statistically tested using log-rank test.

## Results

### Functional genetic dropout CRISPR screens with 3D culture conditions identify novel signals regulate PARPi sensitivity

**Figure 1:**
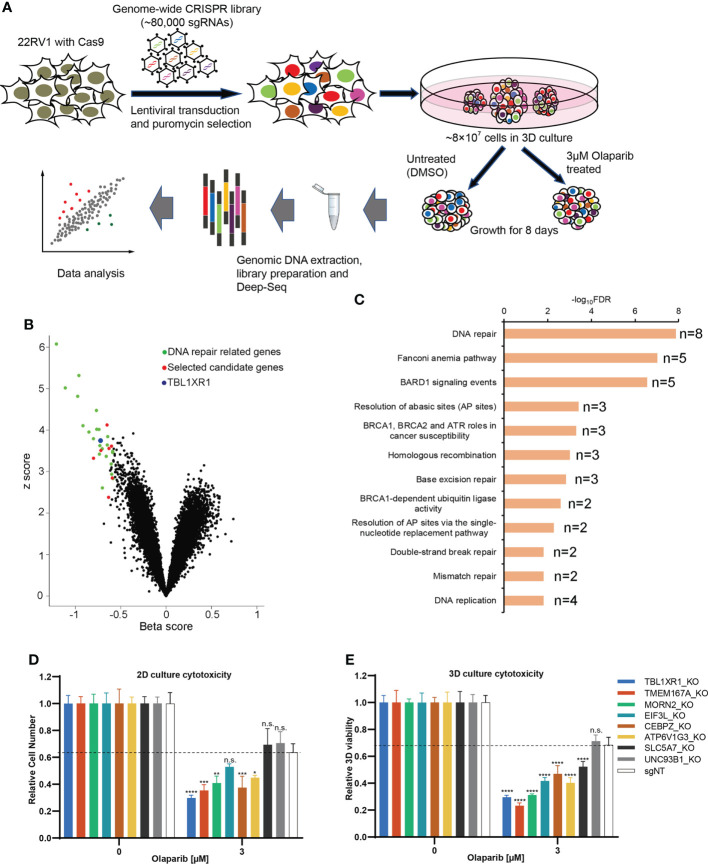
CRISPR screen of prostate cancer line in 3D culture. **(A)** The diagram of steps involved in CRISPR screen in 3D culture. **(B)** Volcano plot of CRISPR screening analysis. Values on the x-axis show the beta score of each gene and the y axis shows absolute z score (see Methods). Previously identified DNA repair genes were labeled in green and selected novel candidate genes were labeled in red. TBL1XR1 is labeled in blue. **(C)** Enriched pathways among the top 46 hits (FDR<0.05, beta<-0.2) from negative selection using the BioPlanet 2019 database set of Enrichr (see Methods). **(D, E)** Selected top candidate hits were validated with individual sgRNAs in 2D **(D)** and 3D **(E)** cytotoxicity assays. Data are mean ± s.e.m., n = 3; *p < 0.05, **p < 0.01, ***p < 0.001, and ****p < 0.0001; n.s., not significant, which were calculated by two-sided t-test between the control (sgNT) and gene-targeting sgRNAs.

To identify genes associated with PARPi resistance in prostate cancer cells, we performed CRISPR screen experiments in 22RV1 cell line. Although 22RV1 is a BRCA2-mutated cell line (**Figure S1A**) (41), the IC50 of olaparib in 22RV1was 14.5μM in our hands (**Figure S1C**), which was much higher than that of the previously reported BRCA2-deficient cell lines (IC50 = 57~124nM), and it was comparable to the BRCA2-proficient cell lines (IC50 = 5.7~10.4μM) (42). Therefore, we concluded that 22RV1 is olaparib relatively insensitive. To explore the mechanism underlying PARPi sensitivity in 22RV1 cells, we performed negative selection of CRISPR screen for olaparib, which focused on the depleted sgRNAs (dropout genes) that resulted in cell death (43). To make the experimental results more closely relevant to the *in vivo* setting, we generated an *in vitro* 3D culture system based on methods published by Han et al. (44) and performed CRISPR screen experiments with 22RV1 3D spheroids (**Figure 1A**). 22RV1 cells stably express CAS9 protein (**Figure S1B**) transduced with a human genome-wide lentiviral library were cultured to form spheroids. The spheroids were treated with DMSO or 3μM olaparib, a concentration that slightly inhibited the growth of 22RV1, but was lethal for PARPi-sensitive cells (**Figure S1C**). The results of deep-sequencing and data analysis showed that the sgRNAs of 46 genes were significantly depleted (beta<0, FDR<0.05) and the sgRNAs of 38 genes were enriched (beta>0, FDR<0.05) (See **Supplementary Data 1**). Among these top dropout genes, 27 genes have been reported to be involved in DNA repair or DNA damage response (**Supplementary Data 1**, **Figure 1B**) (45). Pathway analysis was performed using all significant genes with FDR<0.05 (n=46) and beta<-0.2, and the top12 signaling pathways were all related to DNA repair or DNA replication fork protection (**Figure 1C**). These results indicate that our CRISPR screening results are reliable and biologically relevant. In addition to those reported DNA repair-related genes, we also found genes for which KO significantly affect PARP response and that had not been reported to be associated with DNA repair or DNA replication fork protection, suggesting potential novel function of these genes. Therefore, we selected 8 genes for further functionally validation. Cytotoxicity experiments showed that after knocking out five of the eight candidate genes made cells more sensitive to olaparib, both in 2D and 3D cultures (**Figures 1D, E**; **Figure S1D** and **E**). The proliferation rate of the cells was not affected significantly for any of these genes when knocked out (**Figure S1F**). We focused on TBL1XR1 for further study since this gene showed more significant effect on olaparib sensitivity in both 2D and 3D culture and has been implicated in tumorigenesis and resistance to chemotherapy treatment in several reports (27–32, 46, 47).

### Depletion of TBL1XR1 increases sensitivity to PARPi in prostate cancer cells

**Figure 2:**
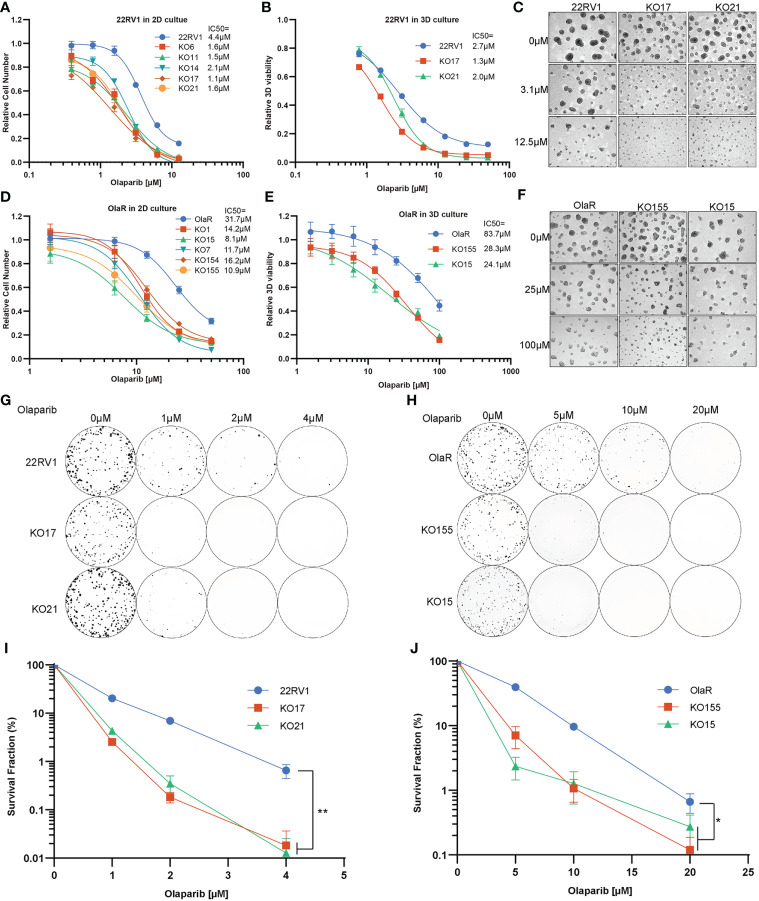
TBL1XR1 deficiency sensitizes prostate cancer cells to PARP inhibitor olaparib. **(A–F)** Increased sensitivity of TBL1XR1 KO cells in 22RV1 **(A–C)** and OlaR **(D–F)** cell lines after treatment with olaparib in 2D **(A, D)** and 3D **(B, E)** culture. Micrographs show images of growth assays for 22RV1 **(C)** and OlaR **(F)** in 3D culture. Data are mean ± s.e.m. normalized to untreated cells. Solid lines show a nonlinear least-squares fit to a four-parameter dose-response model. **(G–J)** Reduced survival of TBL1XR1 KO cells in 22RV1 **(G, I)** and OlaR **(H, J)** cell lines after long-term treatment with olaparib. Data are mean ± s.e.m. normalized to untreated cells. *p < 0.05, **p < 0.01.

To further verify the function of TBL1XR1, we generated monoclonal knockout cell lines of TBL1XR1 (**Figure S2A**). All knockout cell lines were more sensitive to olaparib in 2D culture (**Figure 2A**). We then choose two clones to develop to 3D sphere and performed cytotoxicity assays in 3D culture and the results also showed that KO TBL1XR1 rendered a more sensitive phenotype to olaparib (**Figure 2B**). At higher concentrations (>3μM) of olaparib treatment, the spheroids formed by the knockout cell lines were morphologically smaller and had more debris of dead cells (**Figure 2C**). Our laboratory had generated a 22RV1 olaparib-resistant cell line (OlaR, **Figure S2B**). We also knocked out TBL1XR1 in this olaparib-resistant cell line and generated several monoclonal knockout cell lines (**Figure S2A**). Cytotoxicity assays showed that knockout cell lines in the OlaR background were also more sensitive to olaparib in both 2D and 3D culture (**Figures 2D–F**). To further confirmed the results, we performed clonogenic assays to examine the cell viability of TBL1XR1 knockout cell lines under prolonged treatment with olaparib. In both 22RV1 and OlaR cell backgrounds, TBL1XR1-deficient cell lines were more sensitive to olaparib (**Figures 2G–J**). These results indicate that TBL1XR1 deficiency sensitizes prostate cancer cells to PARPi.

### PARPi sensitization of cells by TBL1XR1 depletion is partially dependent on PARP1

**Figure 3:**
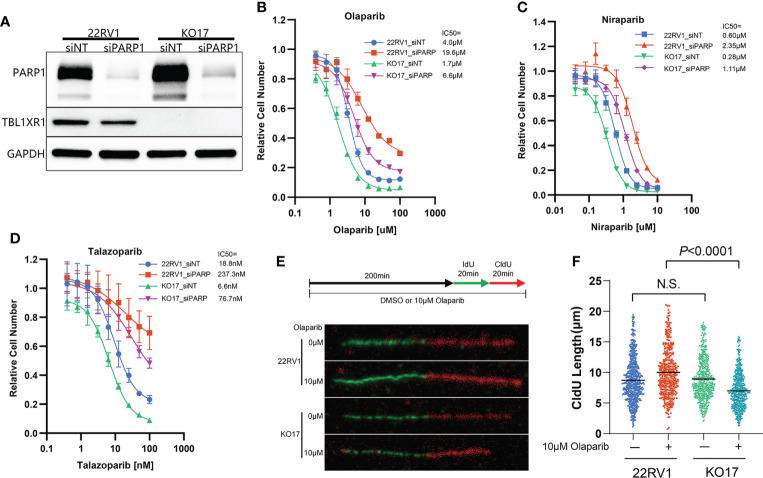
TBL1XR1-deficient sensitizes prostate cancer cells to PARPi partially dependent on PARP trapping and drives the cells to suffer more serious DNA replication stress. **(A–D)** siRNA of PARP1 knockdown PARP1 protein efficiently **(A)** and both WT and TBL1XR1-deficient cells reverse sensitivity to olaparib **(B)**, niraparib **(C)** and talazoparib **(D)** when transfected with PARP1 siRNA. **(E, F)** DNA fiber assays of WT and TBL1XR1-deficient cells under vehicle or 10μM olaparib treatment for 4h (as the diagram in E). Representative images of DNA fibers of different groups are shown in **(E)**. Quantification of red fiber (CldU labeled) length from the ongoing replication was done using the ImageJ software (NIH). At least 200 fibers were measured for each group. *P* values calculated by two-tailed Student’s t-test. N.S. indicated not significant.

The cytotoxicity of PARPi on cells has a similar mechanism, which is driven by PARP trapping (8). To verify that the effect of TBL1XR1 extends to other PARPi, we also used niraparib and talazoparib, which have higher PARP trapping potency than olaparib (3), to perform cytotoxicity experiments. The results showed that TBL1XR1-deficient cells were also more sensitive to niraparib and talazoparib in both 22RV1 parental and OlaR cells (**Figures S2C–F**). To explore whether the sensitization confer from TBL1XR1 deficiency is dependent on PARP function, we knocked down PARP1 (**Figure 3A**) in 22RV1 and TBL1XR1-deficient cells, followed by cytotoxicity assays with PARPi. Compared with cells transfected with control siRNA (siNT), PARP1-knockdown cells exhibited significant resistance to PARPi, both in wild-type (WT) cells and in TBL1XR1-deficient cells (**Figures 3B–D**). However, the TBL1XR1-deficient cells still show more sensitivity than 22RV1 cells even though the PARP1 is dramatically knocked down in both cell lines. These results indicate that the sensitivity of TBL1XR1-deficient cells to PARPi drugs in prostate cancer cells is partially dependent on PARP trapping.

### Depletion of TBL1XR1 increases DNA replication stress but decreases accumulation of γH2AX in cells upon PARPi treatment

**Figure 4:**
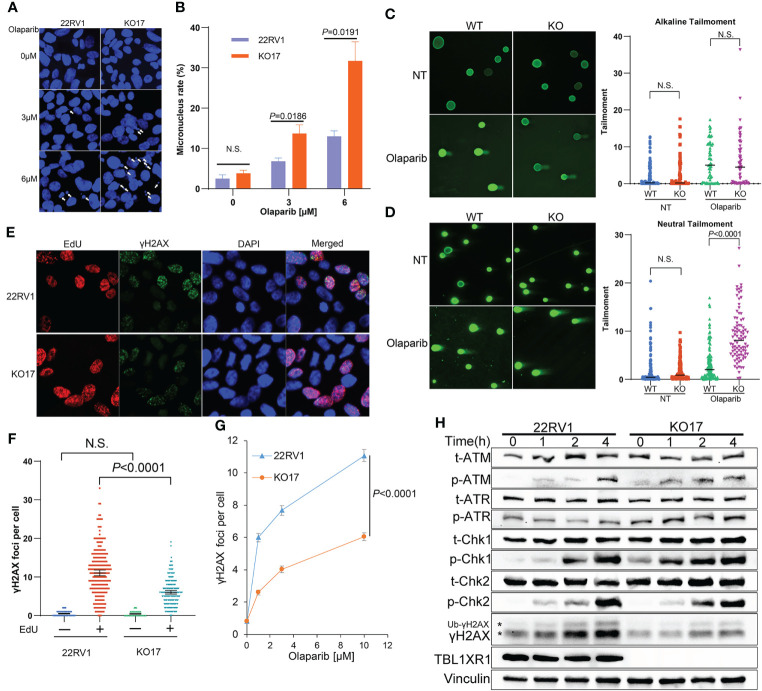
**(A, B)** Micronucleus rate of WT and TBL1XR1-deficient cells under 0μM (vehicle), 3μM or 6μM olaparib treatment for 48h. Micronucleus rate was measured by micronuclei number/total cell nucleus number in one image field **(A)**. 5 image fields were calculated for each experimental group **(B)**. *P* values calculated by two-tailed Student’s t-test. N.S. indicated not significant. **(C, D)** Comet assay of WT and TBL1XR1-deficient cells with or without olaparib treatment in alkaline condition **(C)** and neutral condition **(D)**. NT, no treatment. *P* values calculated by two-tailed Student’s t-test. N.S. indicated non-significant. **(E–G)** γH2AX foci in WT and TBL1XR1-deficient cells under olaparib treatment. Representative images of γH2AX foci and EdU staining in cells after 10μM olaparib treated for 4h were shown in **(E)**. γH2AX foci number per cell in EdU negative or positive cells was calculated by ImageJ software **(F)**. γH2AX foci number per cell in EdU positive cells under 0μM (vehicle), 1μM, 3μM or 10μM olaparib treatment for 4h **(G)**. **(H)** Western blot of DNA damage response proteins in WT and TBL1XR1-deficient cells with 10μM olaparib treatment for 0h (without treatment), 1h, 2h and 4h. * indicated γH2AX and ubiquitied γH2AX (Ub-γH2AX).

PARPi induce DNA replication stress by increasing the speed of fork elongation (48, 49). To explore the role of TBL1XR1 in DNA replication under PARPi stress, we performed a DNA fiber assay. These results showed that the formation of replication fork was slightly accelerated upon olaparib treatment in 22RV1 cells (**Figures 3E, F**), which was consistent with previous reports (23, 48, 49). Whereas in TBL1XR1-deficient cells, the length of CldU decreased upon olaparib treatment (**Figures 3E, F**). This suggested a decrease in the rate of DNA replication or replication fork stalling. These results indicated that TBL1XR1-deficient cells suffered more severe DNA replication stress under PARPi treatment. PARPi-induced DNA replication stress can cause abnormal chromosome segregation, resulting in the formation of micronuclei structures (50, 51). The number of micronuclei in TBL1XR1 knockout cells was significantly increased after 48h treatment with 3μM and 6μM olaparib compared to WT cells (**Figures 4A, B**). This indicated that increased defect in chromosome segregation related to DNA replication stress occurred in TBL1XR1-deficient cells. The comet assays showed that TBL1XR1-deficient cells suffered more DNA damage in neutral condition but not alkaline condition after treated by olaparib (**Figures 4C, D**), suggested that TBL1XR1-deficient cells had more double-strand DNA breaks than WT cells under DNA replication stress.

To study whether TBL1XR1 knockout cells suffer more DNA damage during the DNA replication phase (S phase) upon PARPi treatment, we labeled the S phase of the cell cycle with EdU and performed immunofluorescence staining for γH2AX. As expected, most γH2AX foci occurred in cells during the S phase upon olaparib treatment, suggesting that PARPi drove DNA double-strand breaks (DSB) damage during the S phase of cell cycle (**Figures 4E, F**). Surprisingly, TBL1XR1-deficient cells did not show more γH2AX foci formation, rather, it showed decreased foci compared to WT cells (**Figures 4E–G**). We also detected the proteins (p-ATM, p-ATR, p-Chk1, p-Chk2), that are involved in response to DNA damage in WT cells and TBL1XR1 knockout cells after cells were treated with olaparib for 0, 1, 2, and 4 h. The results showed that the levels of these proteins were similar in WT and TBL1XR1-deficient cells (or slightly increased in TBL1XR1-deficient cells), while the accumulation of γH2AX was significantly decreased in TBL1XR1-deficient cells compared to WT cells (**Figure 4H**). These data suggested that TBL1XR1-deficient cells suffer higher levels of DNA damage under olaparib treatment, but the accumulation of γH2AX in TBL1XR1-deficient cells may be hindered, which prevents DNA damage from being repaired efficiently.

### TBL1XR1 interacts with SMC3 and stabilizes it binding chromatin under replication stress

**Figure 5:**
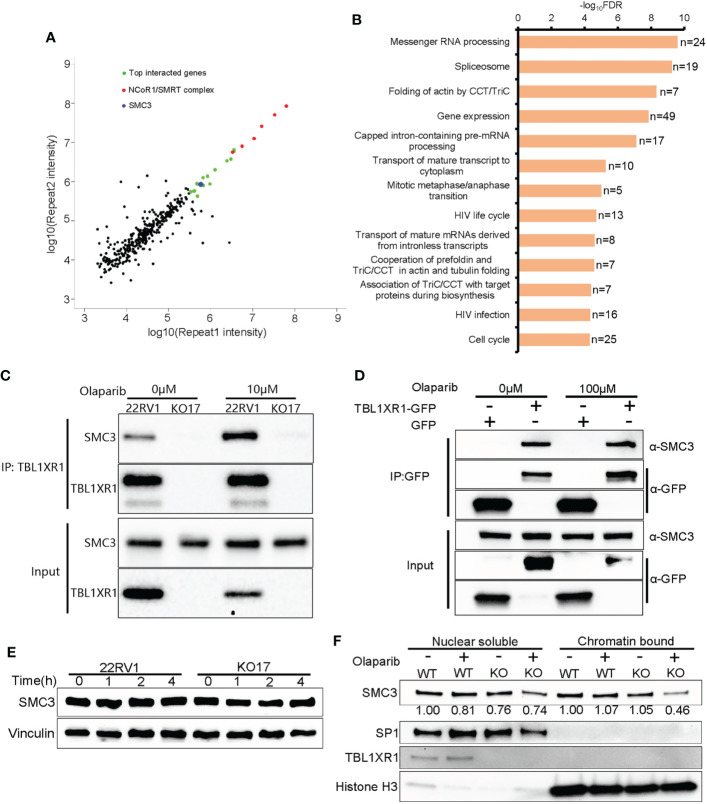
The interaction between TBL1XR1 and SMC3. **(A)** Distribution of IP-MS intensity of the two replicates. **(B)** Pathway analysis of IP-MS signals. **(C, D)** Co-IP validation of interaction between TBL1XR1 and SMC3 in 22RV1 **(C)** and 293T **(D)** cells. **(E)** SMC3 protein levels in 22RV1 and TBL1XR1-deficient cells after 0h, 1h, 2h and 4h treatment of 10μM olaparib. **(F)** Nuclear soluble and chromatin-bound SMC3 protein levels in 22RV1 and TBL1XR1-deficient cells under vehicle or olaparib treatment for 4h. The values of SMC3 protein levels are normalized to SP1 (nuclear soluble) or histone H3 (chromatin-bound) and compared to WT without treatment.

To explore the mechanisms underlying the above phenotypes, we performed immunoprecipitation mass spectrometry (IP-MS) experiments in 22RV1 and TBL1XR1 knockout cells. All peptides that appeared in the WT group but not in the knockout cells group were identified as positive interacted proteins (364 genes in total, see **Supplementary Data 2**). The experimental results indicated that the proteins showing the most significant interactions with TBL1XR1 were all subunits within the NCoR1/SMRT complex (**Figure 5A**). The pathway analysis using all identified interacting proteins indicated that these genes were enriched in the mitosis and cell cycle-related proteins pathways (**Figure 5B**). Among the cohesin complex subunit, SMC3 is one of the most significant candidate (**Figure 5A**). Therefore, we used 22RV1 and TBL1XR1-deficient cells to verify the interaction between TBL1XR1 and SMC3 with and without 10μM olaparib treatment and found that TBL1XR1 interacted with SMC3 in both cases (**Figure 5C**). We also transiently transfected the vector expressing TBL1XR1-GFP protein or empty GFP protein in 293T cells and performed co-IP experiments with or without 100μM olaparib treatment. TBL1XR1-GFP could pull down SMC3 protein under both conditions (**Figure 5D**). These results suggest that the interaction between TBL1XR1 and SMC3 is independent of DNA replication stress. Cohesin is an important complex in regulating mitosis and cell cycle. It has been reported that the deficiency of cohesin complex can sensitize cells to PARPi (52, 53). Recently, Arnould et al. reported that loop extrusion formed by cohesin plays an important role in the spreading of γH2AX on both sides of the DSB sites and the formation of γH2AX foci (33). However, defective TBL1XR1 did not affect the accumulation of total SMC3 (**Figure 5E**). Considering that cohesin needs to bind to chromatin to play a role, we performed cellular fractionation experiments to test whether TBL1XR1 deficiency might affect the amount of SMC3 bound to chromatin. We found that compare to WT, the chromatin bound SMC3 was significantly reduced in TBL1XR1-deficient cells when treated with olaparib (**Figure 5F**). This suggests that TBL1XR1 plays an important role in stabilizing the SMC3 binding to chromatin when cells are under DNA replication stress.

To validate whether SMC3 confers the resistance to PARPi, we performed SMC3 siRNA transfection and cytotoxicity of PARPi in WT 22RV1 cells. The results indicated that knockdown of SMC3 sensitized prostate cancer cells to three PARPi drugs (**Figures 5A–D**). This was consistent with previous reports (52, 53).

### TBL1XR1 and SMC3 are co-participants to confer the resistance to PARPi

**Figure 6:**
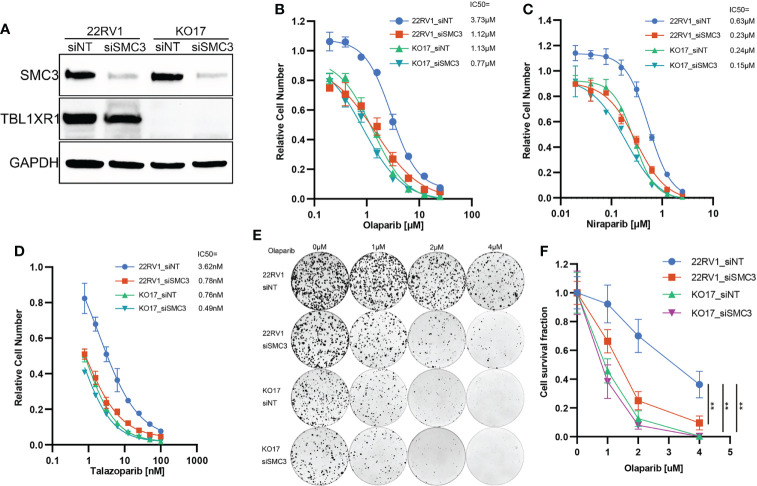
PARPi cytotoxicity and colony formation assay of SMC3 knockdown and TBL1XR1-SMC3 double knockdown (knockout) cells. **(A)** SMC3 knockdown efficiency in 22RV1 and TBL1XR1-knockout cells. **(B–D)** Cytotoxicity of olaparib **(B)**, niraparib **(C)** and talazoparib **(D)** in 22RV1_siNT, 22RV1_siSMC3, KO17_siNT and KO17_siSMC3 cells. siNT, non-target siRNA. **(E, F)** Colony formation assay and cell survival fraction calculation in 22RV1_siNT, 22RV1_siSMC3, KO17_siNT and KO17_siSMC3 cells. siNT, non-target siRNA. **p<0.01.

To validate whether TBL1XR1 and SMC3 work together to confer the resistance to PARPi, we performed SMC3 siRNA transfection and cytotoxicity assay in both 22RV1 and TBL1XR1-deficient cells. The results indicated that the TBL1XR1-SMC3 double knockdown (knockout) did not further sensitize cells to PARPi when compared with either gene knockdown alone (**Figures 6A–D**). The colony formation assays also show similar results (**Figures 6E, F**). These results indicated that TBL1XR1 and SMC3 both have essential functions in the same pathway involved in PARPi sensitivity.

## Discussion

CRISPR Screening technology has been employed to explore genes associated with PARPi drugs susceptibility in human and mouse cell lines (17, 45, 54, 55). In this work, we applied methods from a previous report (44) to generate an inexpensive and large-scale 3D spheroid culture system for a prostate cancer cell line and performed CRISPR screening with olaparib. A scalable 3D spheroid system can be used to enable high-throughput screens that more closely approximate cancers *in vivo* (44). The results of the screening identified novel genes related to PARPi sensitivity that have not been reported before (**Figures 1D, E**), highlighting the advantage of the method.

NCoR1/SMRT nuclear receptor corepressors are among the best-characterized corepressors. In addition to NCoR1 and SMRT (also named NCoR2), this complex also includes HDAC3, TBL1, TBL1XR1 and GPS2. NCoR1/SMRT complex plays important roles in development, metabolism, immunity, etc. through transcriptional regulation (56). HDAC3 has been widely reported to be involved in carcinogenesis (57) and has important functions in the maintenance of chromatin structure, genome stability and DNA damage response (57–59). Here we identified TBL1XR1 as one factor contributing to PARPi sensitivity in prostate cancer cells. We showed that TBL1XR1 helped overcome DNA replication stress by stabilizing SMC3-bound chromatin (**Figure 5F**). Moreover, the TBL1XR1-SMC3 double knockdown (knockout) cells did not further sensitize cells to PARPi compared to either gene knockdown along (**Figure 6**). These data confirmed that both proteins function within the same mechanism.

TBL1XR1-deficient cells suffered increased DNA replication stress when treated with olaparib (**Figures 3E, F**), producing more micronuclei (**Figures 4A, B**), and occurred more double-strand DNA breaks (**Figures 4C, D**). However, the knockout cells have less γH2AX foci accumulation, which is the marker of DNA double-strand breaks (**Figures 4E–H**). These seemingly contradictory data led us to explore possible underlying mechanisms. IP-MS and co-IP experiments revealed the interaction between SMC3 and TBL1XR1 (**Figures 5A–D**). SMC3 is a key subunit of cohesin, which plays an important role in DNA replication and the maintenance of genome stability. It has been previously reported that the absence of cohesin makes cells more sensitive to PARPi through higher levels of DNA replication stress (52, 53, 60). The deficiency of one subunit of the cohesin, STAG2, increases the stalled replication forks (52). Cohesin is critical for the recovery of stalled forks when DNA synthesis is impeded (61). Recent studies have shown that cohesin plays an important role in the spreading of γH2AX near the DSB, and cells lacking cohesin have lower levels of γH2AX accumulation near the DSB (33). We found that although TBL1XR1 did not affect the total amount of SMC3 protein (**Figure 5E**), it could maintain the stability of SMC3-bound chromatin under DNA replication stress (**Figure 5F**). In conclusion, when TBL1XR1-deficient cells were treated with PARPi, the amount of SMC3 bound to chromatin was reduced, resulting in less efficient spreading rate of γH2AX along chromatin near the sites of DSBs, which in turn prevented effective DNA repair, and resulted in higher levels of DNA replication stress and abnormal chromosomal segregation. Deeper mechanistic understanding of the relationships between TBL1XR1 and SMC3, for example, domain specific interactions and whether this interaction is dependent on post-translational modifications require further studies in the future.

## Data availability statement

The original contributions presented in the study are included in the article/**Supplementary Materials**. Further inquiries can be directed to the corresponding author.

## Author contributions

HZ, HG, YG, AJ, and AA conducted experiments. JY, RW and LW supervised experiments. HZ, HG, YG, LWei, and MH analyzed the data. HZ, HG, YG, LWe, JY, and LWang contributed conception and design of the study. HZ, HG, and LWei wrote the manuscript. All authors contributed to the article and approved the submitted version.

## Funding

This work was supported in part by National Institutes of Health (U19 GM61388 to LWa and RW, R01 GM28157 to LW and R01 GM125633 to LWa), Department of Defense (W81XWH-20-1-0262-01 to LWa), Mayo Clinic Centre for Individualized Medicine (MC1351 to LWa), A. T. Suharya and Ghan D. H, Gail and Joseph Gassner, Mayo Clinic Schulze Cancer for Novel Therapeutics in Cancer Research (to LWa).

## Conflict of interest

LWa and RW are cofounders and stock holders of OneOme LLC.

The remaining authors declare that the research was conducted in the absence of any commercial or financial relationships that could be construed as a potential conflict of interest.

## Publisher’s note

All claims expressed in this article are solely those of the authors and do not necessarily represent those of their affiliated organizations, or those of the publisher, the editors and the reviewers. Any product that may be evaluated in this article, or claim that may be made by its manufacturer, is not guaranteed or endorsed by the publisher.

## Supplementary material

Supplementary Figure 1
**(A)** Sanger sequencing results of two mutations in BRCA2 gene. The p.V1810I mutation is a homozygote and the p.TK3030fs is a heterozygote, one allele is WT and another one has a A base insertion. **(B)** Western blot of CAS9 protein in 22RV1 cells. **(C)** Cytotoxicity of olaparib in parental 22RV1 and 22RV1_CAS9 cells. **(D)** Sensitivity in cells with knock out of candidate genes to various doses of olaparib in 2D culture condition. **(E)** Sensitivity in cells with knock out of candidate genes to various doses of olaparib in 3D culture condition. **(F)** Proliferation rate of cells with candidate genes KO. Data are mean ± s.e.m., n = 3; *p < 0.05, **p < 0.01, ***p < 0.001, and ****p < 0.0001; n.s., not significant, which were calculated by two-sided t-test between the control (sgNT) and gene-targeting sgRNAs.

Supplementary Figure 2
**(A)** Western blot of TBL1XR1 in parental 22RV1 (lane No. 1) and OlaR (lane No.7) cells, and corresponding monoclonal knockout cell lines. Lane 2 to 6 are monoclonal knockout cell lines of parental 22RV1. Lane 8 to 12 are monoclonal knockout cell lines of OlaR. **(B)** Cytotoxicity of olaparib in parental 22RV1 and OlaR cells. **(C)** Cytotoxicity of niraparib in parental 22RV1 and corresponding TBL1XR1-deficient cells. **(D)** Cytotoxicity of niraparib in parental OlaR and corresponding TBL1XR1-deficient cells. **(E)** Cytotoxicity of talazoparib in parental 22RV1 and corresponding TBL1XR1-deficient cells. **(F)** Cytotoxicity of talazoparib in parental OlaR and corresponding TBL1XR1-deficient cells.
