# Supplementary material for: 3D CRISPR screen in prostate cancer cells reveals PARP inhibitor sensitization through TBL1XR1-SMC3 interaction
Source: Front Oncol. 2022 Nov 29;12:999302. doi: 10.3389/fonc.2022.999302 (PMC9746894; doi:10.3389/fonc.2022.999302)
Supplement: Supplementary Figure 1 — (A) Sanger sequencing results of two mutations in BRCA2 gene. The p.V1810I mutation is a homozygote and the p.TK3030fs is a heterozygote, one allele is WT and another one has a A base insertion. (B) Western blot of CAS9 protein in 22RV1 cells. (C) Cytotoxicity of olaparib in parental 22RV1 and 22RV1_CAS9 cells. (D) Sensitivity in cells with knock out of candidate genes to various doses of olaparib in 2D culture condition. (E) Sensitivity in cells with knock out of candidate genes to various doses of olaparib in 3D culture condition. (F) Proliferation rate of cells with candidate genes KO. Data are mean ± s.e.m., n = 3; *p < 0.05, **p < 0.01, ***p < 0.001, and ****p < 0.0001; n.s., not significant, which were calculated by two-sided t-test between the control (sgNT) and gene-targeting sgRNAs. [file DataSheet_1.zip › Table 3.DOCX]

Supplementary Table 1. Sequence of primers, crRNAs and siRNAs

| **Name** | **Sequence** | **Source** |
| --- | --- | --- |
| **Primers for knocking out candidate genes of dropout CRISPR screen** | | |
| sgNT-F | TGGAAAGGACGAAACACCGTAGCGAACGTGTCCGGCGTGTTTTAGAGCTAGAAATA | IDT |
| sgNT-R | TATTTCTAGCTCTAAAACACGGAGGCTAAGCGTCGCAAAGAGACGTCCGTCTCCGG |  |
| TBL1XR1_F | TGGAAAGGACGAAACACCGCAAGATGTTCCAAGCAACAGTTTTAGAGCTAGAAATA |  |
| TBL1XR1_R | TATTTCTAGCTCTAAAACTGTTTGATGGTCGACCAATAAGAGACGTCCGTCTCCGG |  |
| TMEM167A_F | TGGAAAGGACGAAACACCCAAGGATCGAATATAAGCACGTTTTAGAGCTAGAAATA |  |
| TMEM167A_R | TATTTCTAGCTCTAAAACAGGATCGAATATAAGCACAGAGAGACGTCCGTCTCCGG |  |
| MORN2_F | TGGAAAGGACGAAACACCCCAAATGGGGCAAAGTATACGTTTTAGAGCTAGAAATA |  |
| MORN2_R | TATTTCTAGCTCTAAAACCATTTGGGAATGTGTAAGTCAGAGACGTCCGTCTCCGG |  |
| EIF3L_F | TGGAAAGGACGAAACACCCTGGTAGTAATCTCCTAACAGTTTTAGAGCTAGAAATA |  |
| EIF3L_R | TATTTCTAGCTCTAAAACCCGCTTGTGTATACCTCCAAAGAGACGTCCGTCTCCGG |  |
| CEBPZ_F | TGGAAAGGACGAAACACCACAAAGCAGCTCATGAGCCAGTTTTAGAGCTAGAAATA |  |
| CEBPZ_R | TATTTCTAGCTCTAAAACCGATGACACAATTGCCTTCAAGAGACGTCCGTCTCCGG |  |
| ATP6V1G3_F | TGGAAAGGACGAAACACCAAAGCGATTGAAGCAAGCCAGTTTTAGAGCTAGAAATA |  |
| ATP6V1G3_R | TATTTCTAGCTCTAAAACTAGATTGTTTTAGTCGAAACAGAGACGTCCGTCTCCGG |  |
| SLC5A7_F | TGGAAAGGACGAAACACCCAGGATGTGACAATGCAAAGGTTTTAGAGCTAGAAATA |  |
| SLC5A7_R | TATTTCTAGCTCTAAAACTGTTTGCACGGAACATCTACAGAGACGTCCGTCTCCGG |  |
| UNC93B1_F | TGGAAAGGACGAAACACCACAATGAGGTTTCCGCTCCGGTTTTAGAGCTAGAAATA |  |
| UNC93B1_R | TATTTCTAGCTCTAAAACCCGTGGGCCGGTAAGCGGCTAGAGACGTCCGTCTCCGG |  |
| **crRNAs for knocking out TBL1XR1** | | |
| crRNA1 | CATCAAACAAGGTACCATCC | IDT |
| crRNA2 | AGATAAGCTTGCACAGCAAC |  |
| **Primers for constructing pWPXL-TBL1XR1 overexpress plasmid** | | |
| pWP_TBL1XR1-F | GAGGTTTAAACTACGGGATCCATGAGTATAAGCAGTGATGAGGTCAAC | IDT |
| pWP_TBL1XR1-R | CCGGTAGCGCTAGGACGCGTAATTTCCGAAGGTCTAATACACAAAC |  |
| **siRNAs for knocking down PARP1 and SMC3** | | |
| siGENMONE SMARTpool PARP1 siRNA  M-006656-01 | GAAAGUGUGUUCAACUAAU | Horizon Discovery |
|  | GCAACAAACUGGAACAGAU |  |
|  | GAAGUCAUCGAUAUCUUUA |  |
|  | GAUAGAGCGUGAAGGCGAA |  |
| siGENMONE SMARTpool SMC3 siRNA  M-006834-01 | GAAAGCAUCUCCUUAAUGA | Horizon Discovery |
|  | GAACGGAUCUUUAUGCAAA |  |
|  | GGAAUUAGGGUGUCAUUUA |  |
|  | GAAGAGAGAUUACAUACUC |  |
| siGENOME Non-Targeting Control siRNA #5  D-001210-05-50 | UGGUUUACAUGUCGACUAA | Horizon Discovery |

**Supplementary Table 2. Antibodies information**

| **Products** | **Source** | **Catalog Number** |
| --- | --- | --- |
| TBL1XR1 rabbit monoclonal antibody | Cell Signaling Technology | 74499S |
| TBL1XR1 mouse monoclonal antibody | Santa Cruz Biotechnology | sc-100908 |
| SMC3 rabbit monoclonal antibody | Cell Signaling Technology | 5696S |
| GAPDH rabbit monoclonal antibody | Cell Signaling Technology | 5174S |
| GFP rabbit monoclonal antibody | Cell Signaling Technology | 2956S |
| SP1 rabbit monoclonal antibody | Cell Signaling Technology | 9389S |
| Histone H3 rabbit monoclonal antibody | Cell Signaling Technology | 4499S |
| PARP1 rabbit monoclonal antibody | Cell Signaling Technology | 9532S |
| ATM rabbit monoclonal antibody | Cell Signaling Technology | 2873S |
| Phospho-ATM (Ser1981) rabbit monoclonal antibody | Cell Signaling Technology | 5883S |
| ATR rabbit monoclonal antibody | Cell Signaling Technology | 13934S |
| Phospho-ATR (Ser428) polyclonal antibody | Cell Signaling Technology | 2853S |
| Chk1 mouse monoclonal antibody | Cell Signaling Technology | 2360S |
| Phospho-Chk1 (Ser345) rabbit monoclonal antibody | Cell Signaling Technology | 2348S |
| Chk2 mouse monoclonal antibody | Cell Signaling Technology | 3440S |
| Phospho-Chk2 (Thr68) rabbit monoclonal antibody | Cell Signaling Technology | 2197S |
| Phospho-Histone H2A.X (Ser139) (γH2AX) rabbit monoclonal antibody | Cell Signaling Technology | 9718S |
| Anti-mouse IgG, HRP-linked Antibody | Cell Signaling Technology | 7076S |
| Anti-rabbit IgG, HRP-linked Antibody | Cell Signaling Technology | 7074S |
| β-Actin mouse monoclonal antibody | Cell Signaling Technology | 3700S |
| Cas9 mouse monoclonal antibody | EpiGentek | A-9000-050 |
| Vinculin mouse monoclonal antibody | MilliporeSigma | V9264 |
| Anti-BrdU clone B44 (detects IdU) mouse monoclonal antibody | BD Biosciences | 347580 |
| Anti-BrdU clone BU1/75 (ICR1) (detects CldU) rat monoclonal antibody | Abcam | ab6326 |
| Anti-mouse IgG F(ab')2 Alexa Fluor 488 conjugate antibody | Cell Signaling Technology | 4408S |
| Anti-rat IgG Alexa Fluor 594 conjugate antibody | Thermo Fisher Scientific | A-11007 |
