# Supplementary figures and images for: 3D CRISPR screen in prostate cancer cells reveals PARP inhibitor sensitization through TBL1XR1-SMC3 interaction
Source: Front Oncol. 2022 Nov 29;12:999302. doi: 10.3389/fonc.2022.999302 (PMC9746894; doi:10.3389/fonc.2022.999302)

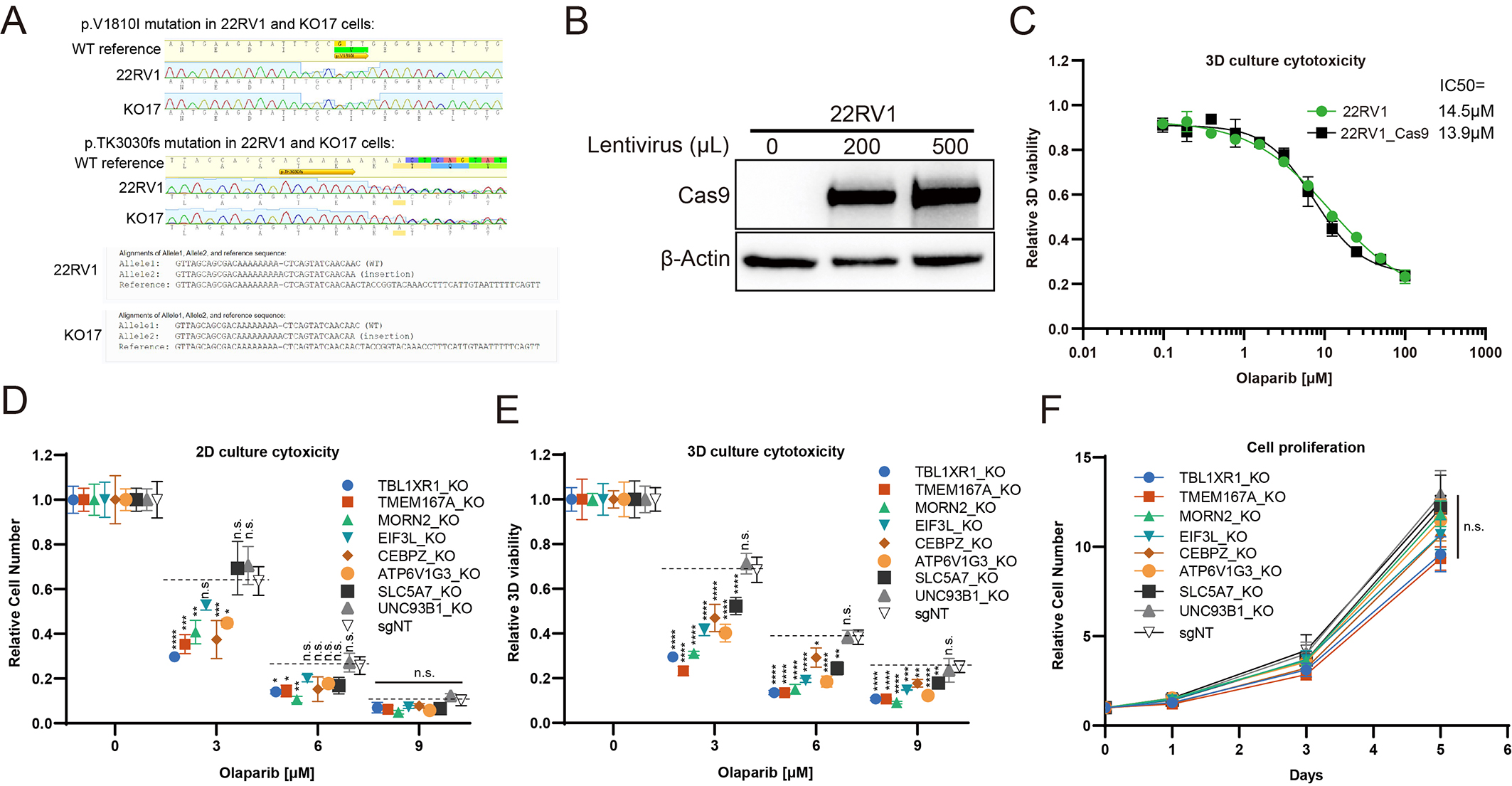

Supplement: Supplementary Figure 1 — (A) Sanger sequencing results of two mutations in BRCA2 gene. The p.V1810I mutation is a homozygote and the p.TK3030fs is a heterozygote, one allele is WT and another one has a A base insertion. (B) Western blot of CAS9 protein in 22RV1 cells. (C) Cytotoxicity of olaparib in parental 22RV1 and 22RV1_CAS9 cells. (D) Sensitivity in cells with knock out of candidate genes to various doses of olaparib in 2D culture condition. (E) Sensitivity in cells with knock out of candidate genes to various doses of olaparib in 3D culture condition. (F) Proliferation rate of cells with candidate genes KO. Data are mean ± s.e.m., n = 3; *p < 0.05, **p < 0.01, ***p < 0.001, and ****p < 0.0001; n.s., not significant, which were calculated by two-sided t-test between the control (sgNT) and gene-targeting sgRNAs. [file DataSheet_1.zip › Image 1.JPEG]

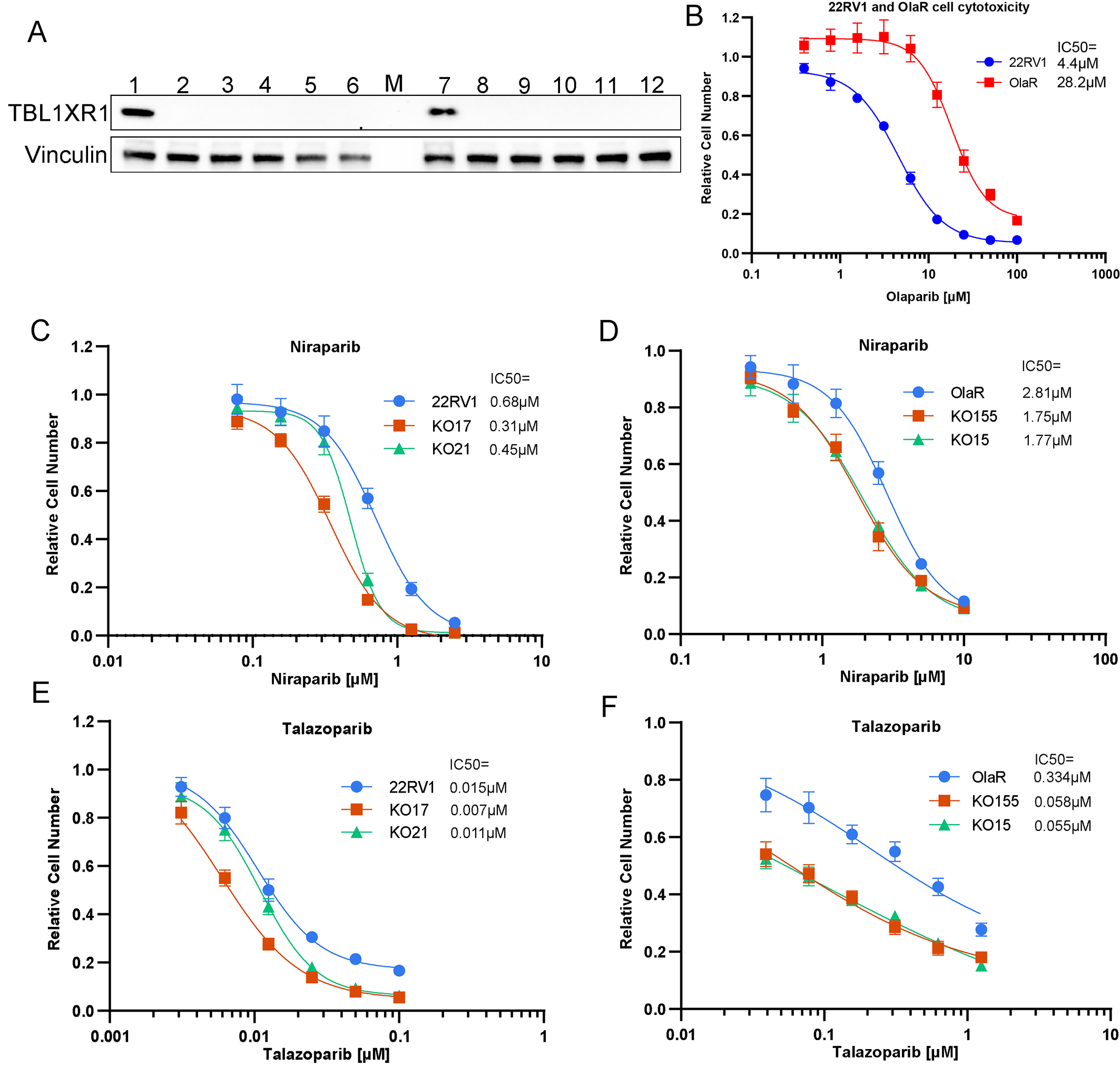

Supplement: Supplementary Figure 1 — (A) Sanger sequencing results of two mutations in BRCA2 gene. The p.V1810I mutation is a homozygote and the p.TK3030fs is a heterozygote, one allele is WT and another one has a A base insertion. (B) Western blot of CAS9 protein in 22RV1 cells. (C) Cytotoxicity of olaparib in parental 22RV1 and 22RV1_CAS9 cells. (D) Sensitivity in cells with knock out of candidate genes to various doses of olaparib in 2D culture condition. (E) Sensitivity in cells with knock out of candidate genes to various doses of olaparib in 3D culture condition. (F) Proliferation rate of cells with candidate genes KO. Data are mean ± s.e.m., n = 3; *p < 0.05, **p < 0.01, ***p < 0.001, and ****p < 0.0001; n.s., not significant, which were calculated by two-sided t-test between the control (sgNT) and gene-targeting sgRNAs. [file DataSheet_1.zip › Image 2.JPEG]
